# A clinical nomogram for evaluating occult central lymph node metastasis in clinically low-risk, unifocal papillary thyroid microcarcinoma

**DOI:** 10.3389/fendo.2026.1853172

**Published:** 2026-09-09

**Authors:** Linan Qin, Yunhe Liu, Xiwei Zhang, Song Ni, Wensheng Liu, Hui Huang, Shaoyan Liu

**Affiliations:** Department of Head and Neck Surgical Oncology, National Cancer Centre/National Clinical Research Centre for Cancer/Cancer Hospital, Chinese Academy of Medical Sciences and Peking Union Medical College, Beijing, China

**Keywords:** central lymph node metastasis (CLNM), nomogram, papillary thyroid microcarcinoma (PTMC), predictive model, risk factors

## Abstract

**Background:**

This study aimed to develop and validate a clinical nomogram to predict the risk of central lymph node metastasis (CLNM) in patients with clinically low-risk papillary thyroid microcarcinoma (PTMC).

**Methods:**

Patients with clinically low-risk PTMC were enrolled in this retrospective cohort study. Potential predictive factors include sex, age, tumor size, tumor location, and Hashimoto’s thyroiditis (HT). Independent predictive factors were identified using multivariate logistic regression and a nomogram was developed using the finalized model. The performance of the model was evaluated using receiver operating characteristic (ROC) curve analysis, calibration plots, and decision curve analysis (DCA).

**Results:**

This study included 4,201 patients with clinically low-risk unifocal PTMC, of whom 1,553 (37.0%) presented with CLNM. Multivariate logistic regression analysis revealed four independent predictive factors: sex, age, tumor size, and tumor location. The constructed nomogram demonstrated moderate predictive performance, with an area under the ROC curve (AUC) of 0.700 (95% confidence interval [CI]: 0.680–0.719) in the training cohort and 0.697 (95% CI: 0.667–0.727) in the validation cohort. Furthermore, the fit of the calibration curves was good, and the DCA results suggest potential clinical utility for patient benefit.

**Conclusion:**

A clinical nomogram incorporating sex, age, tumor size, and tumor location was developed and internally validated. This tool achieved moderate predictive accuracy for estimating the risk of CLNM in patients with clinically low-risk unifocal PTMC. This screening-level model may provide auxiliary clinical value for outpatient consultation. It can aid clinicians in patient counselling and facilitate preliminary risk stratification and clinician–patient communication for this patient population.

## Introduction

The increasing incidence of papillary thyroid carcinoma (PTC) in recent decades is largely attributable to the increased detection of papillary thyroid microcarcinoma (PTMC) (1). According to the American Thyroid Association (ATA) guidelines, patients with PTMC are considered clinically low-risk if they meet the following criteria: cT1N0 staging, unifocal tumor, no ultrasonographic evidence of extrathyroidal extension, no cytological or molecular (if performed) findings suggestive of aggressive disease, no family history of thyroid cancer, and no prior history of neck radiation therapy (2). Contemporary clinical guidelines delineate several acceptable management options for patients diagnosed with clinically low-risk PTMC, including traditional surgery, active surveillance (AS), and radiofrequency ablation (RFA) (3). AS has been established as a safe and dependable strategy, with extensive research demonstrating long-term oncological outcomes that are non-inferior to those achieved with immediate surgery (4, 5). Concurrently, RFA has gained substantial recognition as a primary therapeutic option for patients with T1aN0M0 PTMC (3). Its application is expanding, with current expert consensus indicating that RFA is a viable alternative, even for certain tumors that exhibit slightly more advanced characteristics (6).

Therefore, we conducted a retrospective analysis of the data from patients with clinically low-risk PTMC who underwent surgical treatment. Our aim was to assess the risk of occult CLNM using clinical features and demographic data, construct a prediction model, and enable expedited risk stratification and serve as an auxiliary tool to facilitate preliminary risk stratification and clinician–patient communication during outpatient consultations.

## Materials and methods

### Patients

**Figure 1:**
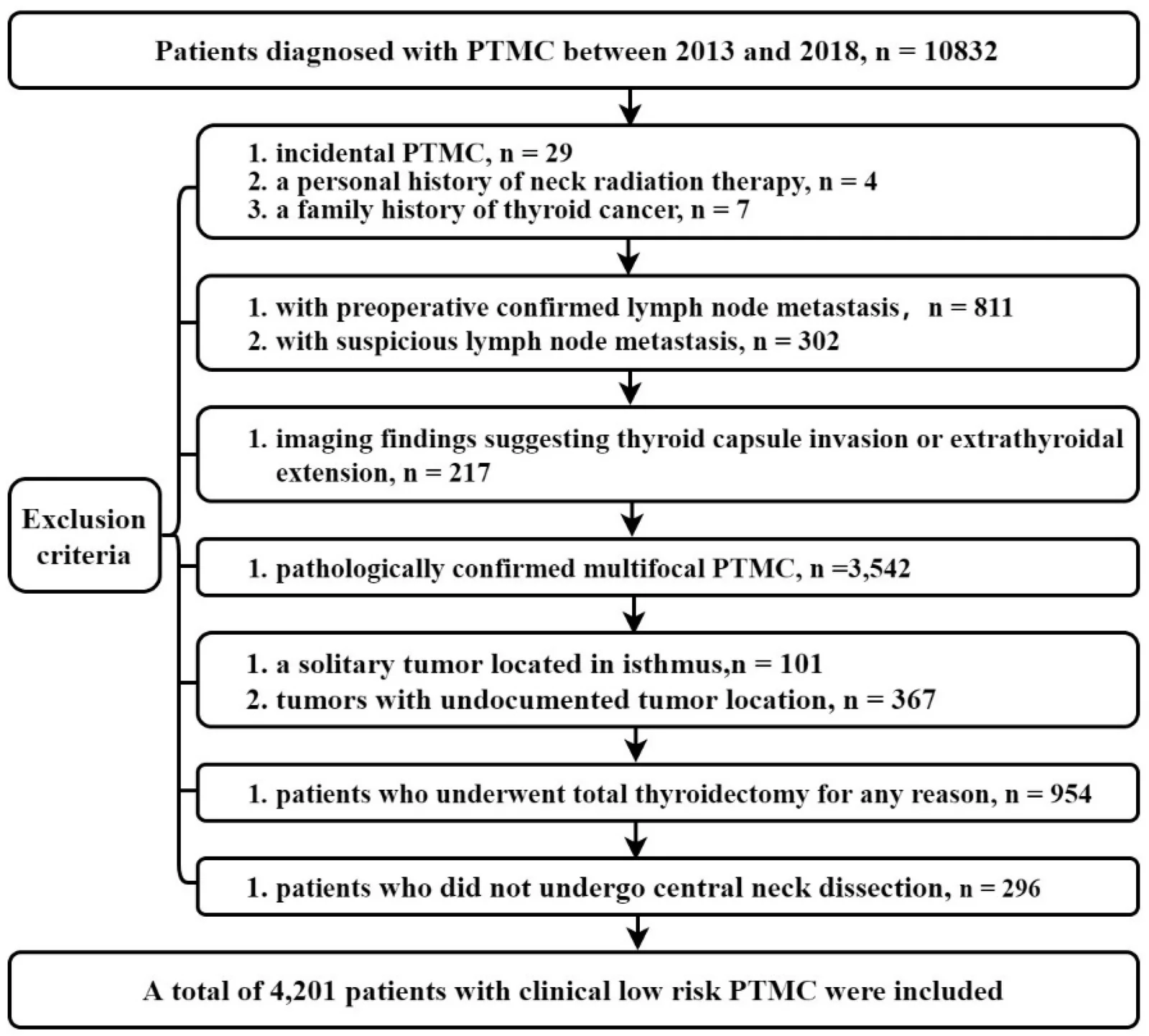
The patient selection flowchart.

This retrospective cohort study was performed at a single institution. We analyzed a cohort of consecutive patients who underwent surgery for PTMC between January 2013 and December 2018. This study adhered to the Strengthening the Reporting of Observational Studies in Epidemiology (STROBE) guidelines. This retrospective analysis was approved by the Ethics Committee of the institution, and written informed consent was obtained from all patients at the time of surgery for the use of clinical data. Patients were excluded based on the following criteria: incidental PTMC; personal history of neck radiation therapy; family history of thyroid cancer; preoperative imaging evidence of confirmed or suspicious lymph node metastasis in the central or lateral neck compartments; imaging findings suggesting thyroid capsule invasion or extrathyroidal extension; pathologically confirmed multifocal PTMC; tumors located in the isthmus or with an undocumented tumor location; total thyroidectomy for any reason; and no central neck dissection. A patient selection flowchart is presented in **Figure 1**.

### Data collection

Data, including age, sex, and clinicopathological characteristics, were obtained from medical records. Primary tumor size was determined based on the maximum diameter of the tumor, as measured via preoperative ultrasound (US). Tumor location was defined as follows: upper portion refers to the upper third of the thyroid gland, lower portion refers to the lower third of the thyroid gland, and middle portion refers to the middle third of the thyroid gland. The clinical lymph node status was primarily evaluated using preoperative US (21). Additionally, palpable firm lymph nodes, evidence of lymph node fusion, or identification of invasive lymph nodes during surgery were considered clinical LNMs. Pathological diagnoses were determined based on the standard World Health Organization criteria (22). In this study, pathological characteristics included Hashimoto’s thyroiditis (HT), histological variants, ETE, LVI, perineural invasion (PNI), and lymph node status (including the number of metastatic LNs and ENE). Initial ROR stratification was retrospectively reclassified based on archived clinicopathological data in accordance with the 2025 ATA guidelines (3).

### Statistical analysis

All statistical analyses were performed using R software (version 4.2.2, R Foundation for Statistical Computing, Vienna, Austria). The baseline characteristics of the study cohort are summarized as follows: categorical variables are reported as counts and percentages, while continuous variables are expressed as medians with corresponding interquartile ranges (IQRs). To examine the potential nonlinear relationships between changes in continuous variables (such as age and tumor size) and CLNM, a logistic regression model incorporating restricted cubic splines (RCS) was applied. The global p-value (overall p-value) was used to evaluate the overall significance of the relationship. Furthermore, to determine whether the association deviated from linearity, the p-nonlinear value was examined, where a p-nonlinear < 0.05 indicates a statistically significant nonlinear relationship. The data were then randomly divided into training and validation cohorts at a ratio of 7:3 and the variables were compared. In the univariate analysis, the chi-square test or Fisher’s exact test was used to analyze categorical variables, whereas Student’s t-test or the rank-sum test was used to analyze continuous variables. In the training cohort, least absolute shrinkage and selection operator (LASSO) logistic regression analysis was used for multivariate analysis to screen for independent risk factors and to construct a prediction nomogram for CLNM. The performance of the model was assessed using a receiver operating characteristic (ROC) curve and a calibration curve, with the area under the ROC curve (AUC) ranging from 0.5 (not discriminant) to 1 (completely discriminant). Decision curve analysis (DCA) was performed to determine the net benefit threshold of the prediction. Results with a p value <0.05 were considered significant. All statistical analyses were performed using R software.

## Results

### Demographic and clinicopathological characteristics

A total of 10,832 patients were diagnosed with PTMC, and 4,201 who met the inclusion criteria were included in this study. Among them, 1,059 (25.2%) were male. The median patient age was 42 years (IQR: 35–50 years). The median tumor size was 0.60 cm (IQR: 0.5–0.8 cm). The tumor was located in the upper portion of the thyroid in 948 patients (22.6%), in the middle portion in 2,209 patients (52.6%), and in the lower portion in 1,044 patients (24.9%). A total of 960 patients (22.9%) had HT. Aggressive variants were present in nine patients (0.2%), including high cell (n = 2), diffuse sclerosis (n = 1), and solid (n = 6). LVI and PNI were observed in 20 (0.5%) and 38 (0.9%) patients, respectively. A total of 1,553 (37.0%) patients had pathological central lymph node metastasis (CLNM) (pN1a). A total of 1,685 patients (40.1%) had microscopic ETE (mETE). A total of 115 patients (2.7%) had five or more mLNs. ENE was observed in 126 (3.0%) patients. According to the 2025 ATA ROR stratification system, 2390 patients (56.9%) were classified as low-risk, 1628 patients (38.8%) as low-intermediate risk, 57 patients (1.4%) as intermediate-high risk, and 126 patients (3.0%) as high-risk. Details of these characteristics are presented in **Table 1**.

**Table 1:** Patient baseline demographic and clinicopathological features.

Characteristic	N = 4,201
Male sex, n (%)	1,059 (25.2%)
Age, years, median (IQR)	42 (35, 50)
Tumor size, cm, median (IQR)	0.60 (0.50, 0.80)
Aggressive variant, n (%)	9 (0.2%)
LVI, n (%)	20 (0.5%)
PNI, n (%)	38 (0.9%
HT, n (%)	960 (22.9%)
ETE, n (%)	1,685 (40.1%)
Tumor location, n (%)	
upper	948 (22.6%)
middle	2,209 (52.6%)
lower	1,044 (24.9%)
CLNM, n (%)	1,553 (37.0%)
Number of mLNs, median (IQR)	0.00 (0.00, 1.00)
Hv-LNM, n (%)	115 (2.7%)
ENE, n (%)	126 (3.0%)
ATA ROR stratification, n (%)	
low	2,390 (56.9%)
low-intermediate	1,628 (38.8%)
intermediate-high	57 (1.4%)
high	126 (3.0%)

IQR, interquartile range; LVI, lymphovascular invasion; PNI, perineural invasion; HT, Hashimoto's Thyroiditis; ETE, extrathyroidal extension; CLNM, central lymph node metastasis; mLNs, metastatic lymph node; Hv-LNM, high-volume lymph node metastasis; ENE, extranodal extension; ROR, risk of recurrence.

### The association between age and tumor size and the risk of CLNM

**Figure 2:**
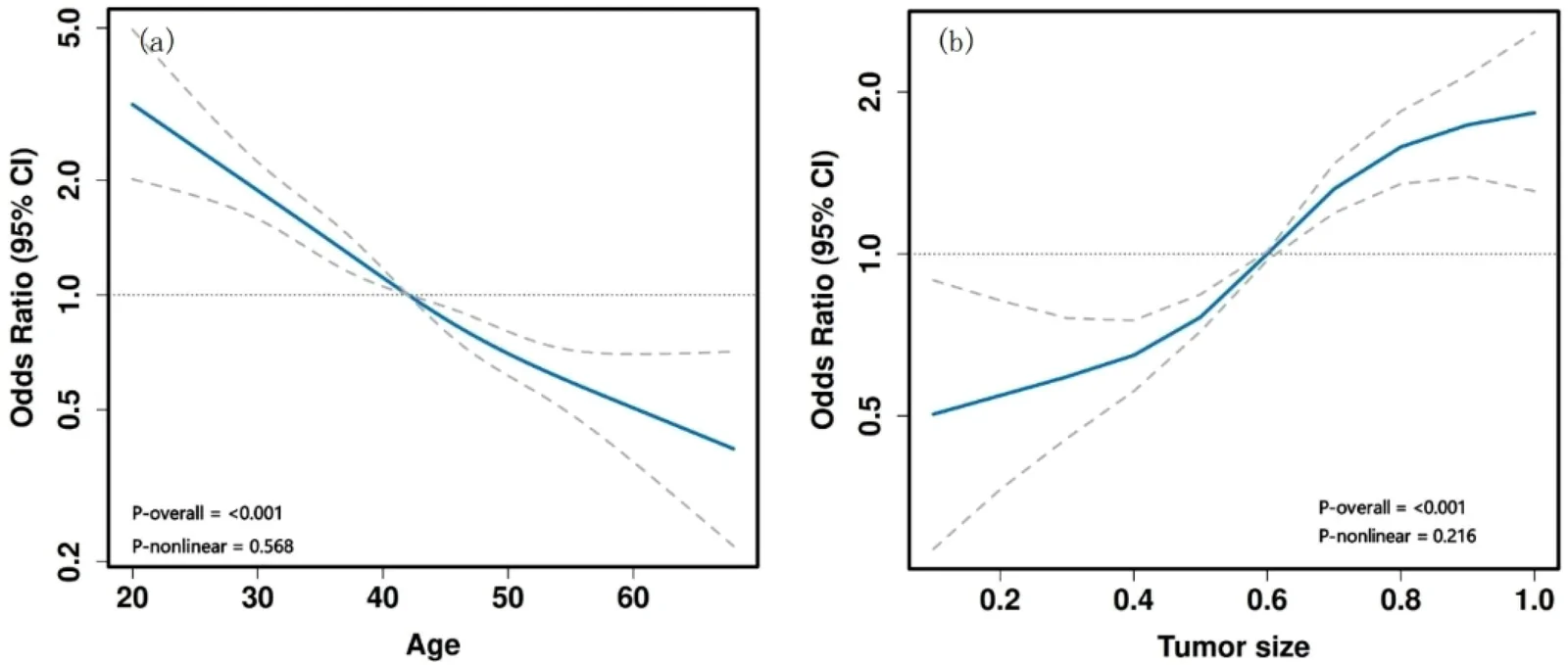
Association between age **(a)** and tumor size **(b)** and CLNM with the RCS function. Model with four knots positioned at the 5th, 35th, 65th, and 95th percentiles. **(a)** Y-axis represents the OR to present CLNM for any value of age compared to individuals with reference value (50th percentile) of age. The logistic regression was adjusted for sex, tumor location, tumor size, and HT. **(b)** Y-axis represents the OR to present CLNM for any value of tumor size compared to individuals with reference value (50th percentile) of age. The logistic regression was adjusted for sex, age, tumor location, and HT.

The RCS within a multivariable logistic regression analysis, adjusted for sex, tumor location, tumor size, and HT, revealed a statistically significant linear relationship between age and CLNM (p-value for overall age effect: < 0.001; p-value for nonlinear component: 0.568). The plotted curve indicated a consistent inverse linear association, demonstrating that advancing age was associated with a progressively lower risk of CLNM (**Figure 2a**). Similarly, the RCS analysis for tumor size, adjusted for sex, age, tumor location, and HT, revealed a significant linear association with CLNM risk (p for overall effect < 0.001; p for nonlinearity = 0.216). The plotted curve illustrated a clear positive linear relationship, confirming that greater tumor size was associated with an elevated risk of CLNM (**Figure 2b**).

### Development of a nomogram model

The training cohort included 2,941 patients, whereas the internal test cohort included 1,260 patients. The baseline demographic and clinical features of the training and internal test cohorts are summarized in **Table 2**. The two cohorts were well balanced across most of the examined variables. HT was observed in 21.8% of the training cohort and in a slightly higher proportion (25.3%) of the internal test cohort, with a statistically significant difference (p=0.013). PNI was present in 0.7% of the training cohort and 1.3% of the internal test cohort, with a statistically significant difference (p=0.046). Univariate analyses were conducted to assess the association between CLNM and sex, age, tumor size, tumor location, and HT (**Table 3**).

**Table 2:** Patient demographics and clinical features in training and internal test cohort.

Characteristic	Cohort		p-value
	Training cohort N = 2,941	Internal test cohort N = 1,260	
Male sex, n (%)	739 (25.1%)	320 (25.4%)	
Age, years, median (IQR)	42 (35, 50)	42 (35, 50)	0.536^2^
Tumor location, n (%)			0.583^1^
upper	652 (22.2%)	296 (23.5%)	
middle	1,549 (52.7%)	660 (52.4%)	
lower	740 (25.2%)	304 (24.1%)	
Tumor size, cm, median (IQR)	0.60 (0.50, 0.80)	0.60 (0.50, 0.80)	0.266^2^
Aggressive variant, n (%)	6 (0.2%)	3 (0.2%)	>0.999^3^
LVI, n (%)	14 (0.5%)	6 (0.5%)	>0.999^1^
PNI, n (%)	21 (0.7%)	17 (1.3%)	0.046^1^
HT, n (%)	641 (21.8%)	319 (25.3%)	0.013^1^
ETE, n (%)	1,176 (40.0%)	509 (40.4%)	0.804^1^

^1^ Pearson's Chi-squared test, ^2^Wilcoxon rank sum test, ^3^Fisher's exact test.

IQR, interquartile range; LVI, lymphovascular invasion; PNI, perineural invasion; HT, Hashimoto's Thyroiditis; ETE, extrathyroidal extension.

**Table 3:** Univariate analyses of the factors for CLNM in training and internal test cohort.

Characteristic	Training cohort, N = 2941			Internal test cohort, N = 1260		
	No CLNM N = 1,862	CLNM N = 1,079	p-value^1^	No CLNM N = 786	CLNM N = 474	p-value^1^
Male sex, n (%)	385 (20.7%)	354 (32.8%)	<0.001	163 (20.7%)	157 (33.1%)	<0.001
Age, years, median (IQR)	44 (37, 51)	38 (32, 46)	<0.001	44 (37, 51)	38 (32, 46)	<0.001
Tumor location, n (%)			<0.001			<0.001
upper	487 (26.2%)	165 (15.3%)		220 (28.0%)	76 (16.0%)	
middle	990 (53.2%)	559 (51.8%)		400 (50.9%)	260 (54.9%)	
lower	385 (20.7%)	355 (32.9%)		166 (21.1%)	138 (29.1%)	
Tumor size, cm, median (IQR)	0.60 (0.50, 0.70)	0.70 (0.50, 0.80)	<0.001	0.60 (0.40, 0.70)	0.70 (0.50, 0.80)	<0.001
HT, n (%)	432 (23.2%)	209 (19.4%)	0.015	220 (28.0%)	99 (20.9%)	0.005

^1^ Pearson's Chi-squared test; Wilcoxon rank sum test.

IQR, interquartile range; HT, Hashimoto's Thyroiditis; CLNM, central lymph node metastasis.

**Figure 3:**
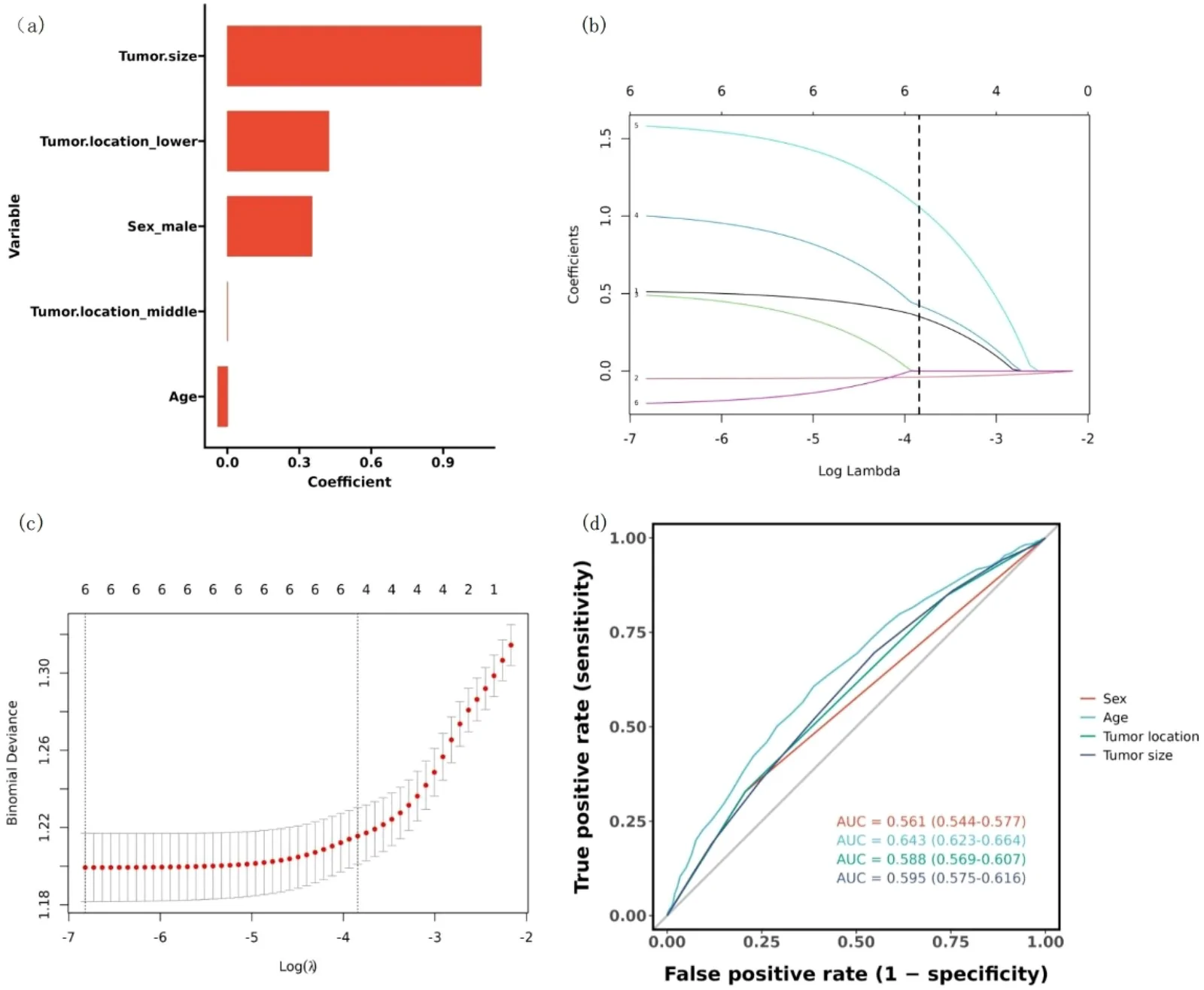
**(a)**Lasso-selected predictors and corresponding coefficients. Only variables with non-zero coefficients after Lasso selection are shown. **(b)** Plot of the Lasso regression coefficient profiles (λ = 0.0214338520878515). **(c)** 10-fold cross-validation was applied to select the most suitable variable using the Lasso regression model. (λ = 0.0214338520878515). **(d)** Comparison of Receiver operating characteristic (ROC) Curves for Individual Predictive Variables. ROC curves for variables assessed in univariate logistic regression.

The candidate variables, sex, age, tumor location, tumor size, and HT, were included in the original model and subsequently reduced to four potential predictors using LASSO regression analysis performed in the training cohort. The coefficients are listed in detail in **Table 4** and the coefficient profiles are plotted in **Figures 3a, b**. A cross-validated error plot of the LASSO regression model is shown in **Figure 3c**. The most regularized and parsimonious model, with a cross-validated error within one standard error of the minimum, included four variables. Receiver operating characteristic (ROC) curve analysis was performed to evaluate the predictive performance of each variable for CLNM risk. As shown in **Figure 3d**, ROC analysis of the abovementioned variables yielded AUC values greater than 0.5.

**Table 4:** The coefficients of Lasso regression analysis.

Variable	Coefficient
(Intercept)	0.24265048
Sex_male	0.35199123
Age	-0.03945427
Tumor location_middle	0.00000000
Tumor location_lower	0.42256149
Tumor size	1.05851055
HT_present	0.00000000

HT, Hashimoto's Thyroiditis.

**Figure 4:**
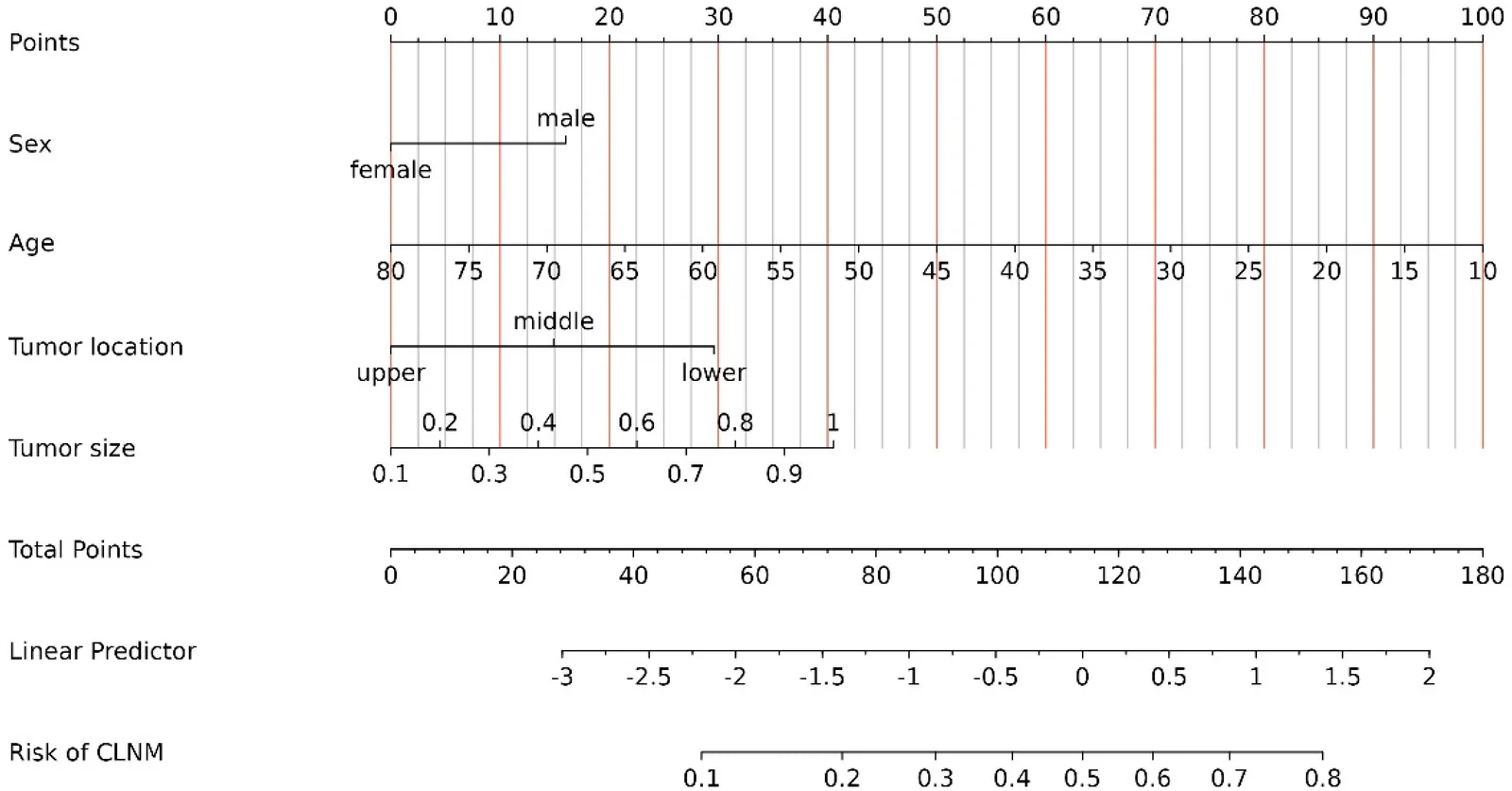
Nomogram prediction model for CLNM.

Multivariate logistic regression analysis of the training cohort revealed four independent predictors of CLNM risk. Compared to female sex, male sex was significantly associated with an increased risk of CLNM (OR 1.75, 95% CI 1.46 to 2.09; p < 0.001). Increased age was associated with a decreased risk of CLNM (OR 0.95, 95% CI 0.94 0.96; p < 0.001). In terms of tumor location, tumors in the middle portion (OR 1.68, 95% CI 1.36 to 2.09; p < 0.001) and lower portion (OR 2.82, 95% CI 2.22 to 3.57; p < 0.001) were associated with a significantly greater risk of CLNM than those in the upper portion. Larger tumor size was also a strong independent risk factor for CLNM (OR 4.84, 95% CI 3.26 to 7.18; p < 0.001). These four independent predictors were subsequently incorporated into the development of a clinical nomogram, as illustrated in **Figure 4**.

### Validation of the nomogram

**Figure 5:**
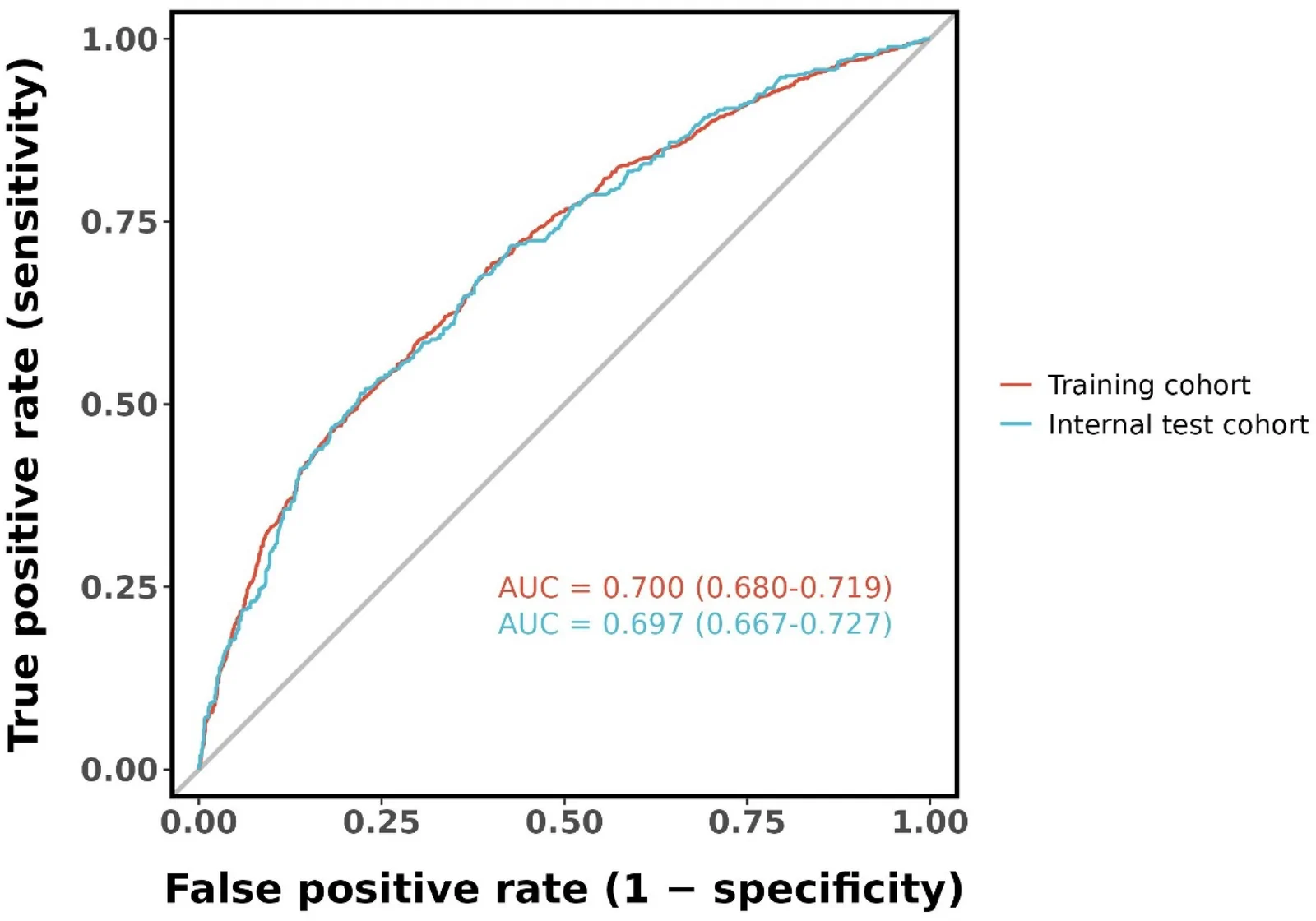
ROC curve of the nomogram.

**Figure 6:**
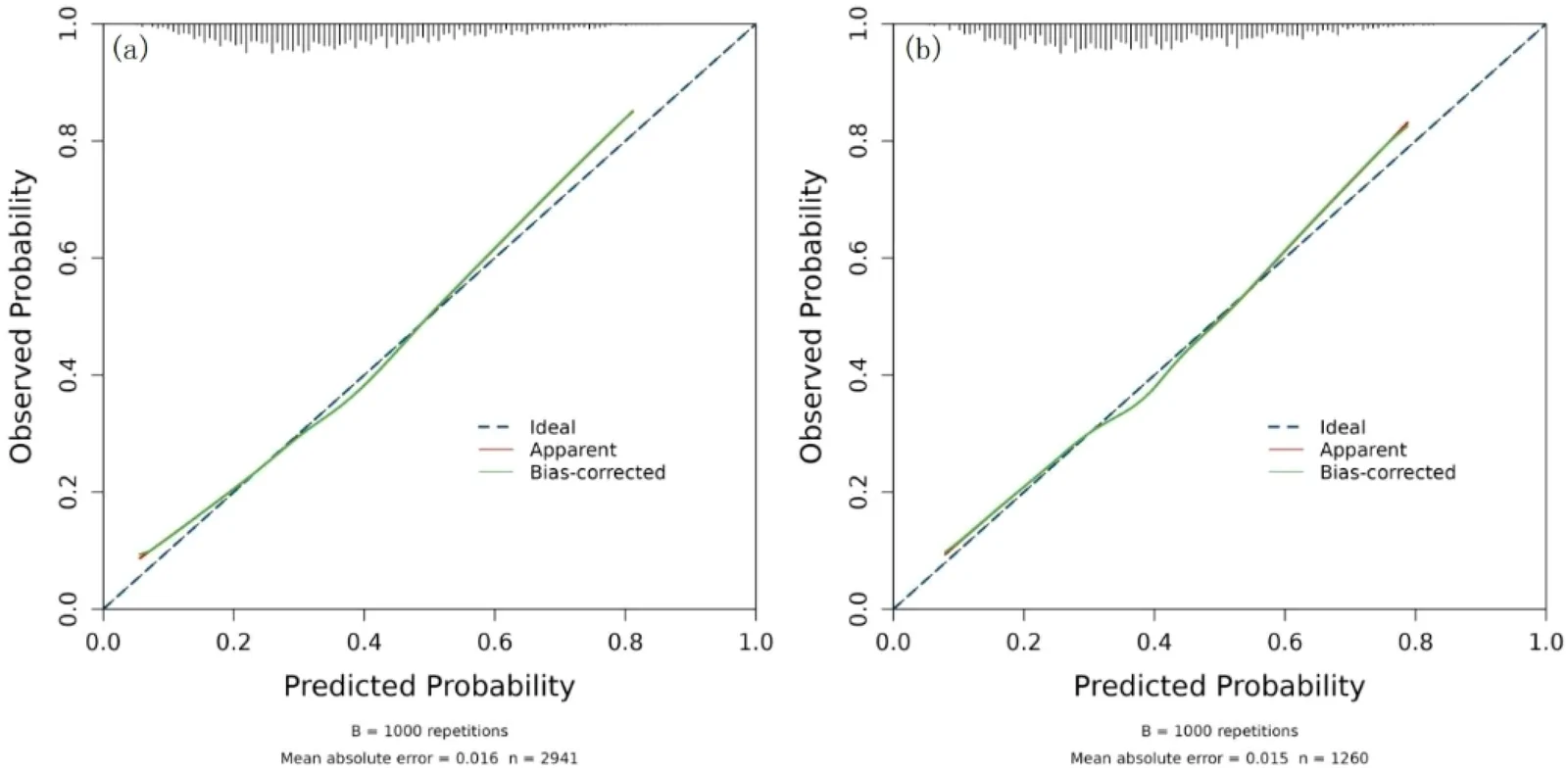
Calibration curve of the nomogram prediction model. **(a)** In the training cohort; **(b)** in the internal validation cohort.

**Figure 7:**
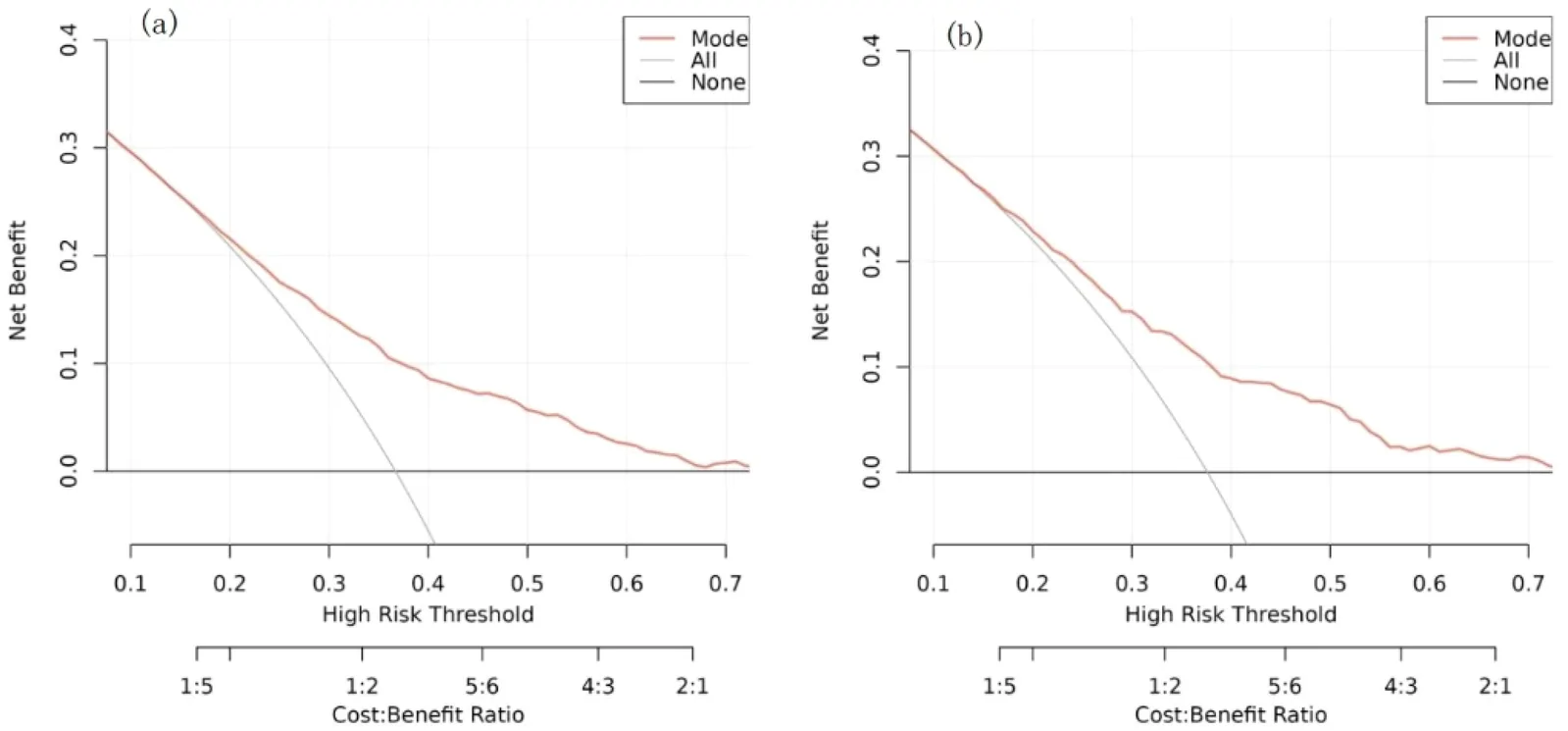
Decision curve analysis of the nomogram. **(a)** In the training cohort; **(b)** in the internal validation cohort.

Receiver operating characteristic (ROC) curve analysis of the CLNM prediction model revealed that, in the training cohort, the area under the curve (AUC) was 0.700 (95% CI: 0.680–0.719), indicating moderate discriminative ability. Performance was maintained in the independent validation cohort, yielding a comparable AUC of 0.697 (95% CI: 0.667–0.727) (**Figure 5**). The calibration plot derived from the bootstrap samples showed excellent concordance between the predicted probabilities of CLNM and the actual observed CLNM rate, as the calibration curve closely followed the ideal 45-degree reference line (**Figure 6**). DCA applied to the nomogram revealed that, over a threshold probability range of 0.1 to 0.7, the net benefit curve of the nomogram remained consistently higher than that of both the “treat-all” and “treat-none” reference lines (**Figure 7**). These results support the potential clinical utility of this nomogram as a screening-level tool for practical implementation.

## Discussion

Occult lymph node metastasis (LNM) is a primary factor influencing the selection of treatment strategies. For clinically node-negative (cN0) PTMC, the rate of central lymph node metastasis (CLNM) ranges from 15.3% to 49.2% (7). Consequently, a significant clinical limitation of nonsurgical treatment approaches is their inability to address occult CLNM. Studies indicate that the number of CLNMs, particularly more than five, is a risk factor for lateral neck lymph node metastasis and regional recurrence (8, 9). Additionally, extranodal extension (ENE) is recognized as a high-risk factor for recurrence and poor prognosis (10, 11), and is consequently considered a significant feature for the high-risk group within the 2025 ATA risk of recurrence (ROR) stratification system (3). The results of our previous study indicated that a significant proportion of patients with clinically low-risk PTMC exhibit high-risk pathological characteristics. For instance, 139 patients (2.8%) presented with five or more metastatic lymph nodes (mLNs), whereas 140 patients (2.8%) presented with ENE (12). These findings underscore the need to consider occult CLNM when selecting appropriate treatment strategies. Furthermore, preoperative evaluation of cervical lymph node status primarily depends on ultrasonography. However, owing to limitations in imaging technology and variability in operator expertise, ultrasound frequently fails to detect metastases. The reported sensitivity for identifying abnormal cervical lymph nodes is only 25–60% (13), with a particularly low diagnostic sensitivity for CLNM (14). The management of prophylactic central neck dissection (pCND) has evolved into a more selective and conservative strategy in contemporary clinical practice (3). However, pCND remain particularly significant in high-risk patients. This procedure can effectively eradicate microscopic metastases that might otherwise go undetected, potentially reducing local recurrence rates (15). Extensive research has been dedicated to assessing the risk of occult CLNM in patients with cN0 PTMC and to the development of corresponding predictive models. Potential risk factors for occult CLNM include sex, age, tumor size, multifocality, extrathyroidal extension (ETE), lymphovascular invasion (LVI), microcalcifications, and some molecular biomarkers (7, 16–20). Although these models typically demonstrate a strong predictive performance, they frequently depend on pathological findings and molecular biomarkers. This dependency restricts their practical application in guiding treatment decisions, particularly in outpatient settings, because of their computational complexity and cost, which hinder accessibility and efficiency in routine clinical practice.

This study revealed four independent risk factors associated with CLNM in patients with clinically low-risk PTMC: sex, age, tumor size, and tumor location. A predictive model was developed and validated by using these factors. AUC confirmed the diagnostic value of the model. Furthermore, decision curve analysis visually demonstrated its utility in supporting clinical decision-making. In outpatient settings, this model can be applied to estimate the risk of occult CLNM, thereby offering a valuable foundation for preliminary risk stratification and clinician–patient communication.

In the present study, the occult CLNM rate was 37.0%, which is consistent with the findings of previous studies, ranging from 15.3% to 49.2% (7). The research approach of this study is grounded in the need to consider the risk of occult CLNM when selecting treatment options for clinically low-risk PTMC; thus, we employed stringent selection criteria. Patients with multifocal tumors, solitary isthmic tumors, preoperative ultrasonographic evidence of an ill-defined margin between the tumor and the thyroid capsule, or suspected ETE were excluded. Based on the current evidence in the literature, ETE and multifocality have been established as significant predictors of CLNM (7, 17), and patients with tumors located in the isthmus also have an elevated risk of CLNM (20, 23). Although certain clinical guidelines acknowledge radiofrequency ablation (RFA) as a feasible therapeutic option for isthmus-located tumors, multifocal tumors (with up to three foci), and PTMC with minimal ETE (6), our institutional approach has remained conservative, prioritizing surgical resection in these scenarios. Consequently, the present analysis was confined specifically to solitary clinically low-risk PTMCs.

Several predictive models have been developed for CLNM in patients with cN0 PTMC. Key factors consistently integrated into these models include patient sex, age, tumor size, multifocality, ETE, and LVI (7, 16–18). Furthermore, several ultrasonographic features, such as the proximity of the tumor to the thyroid capsule and presence of microcalcifications, are frequently incorporated as candidate predictive variables (16–18, 20). A recent model was developed that integrates key preoperative hematological biomarkers, specifically the serum concentrations of albumin, apolipoprotein B, and thyroglobulin (17). Research has progressed with the inclusion of molecular biomarkers. A notable model introduced by Wang et al. was based on the expression levels of three genes: Fibronectin 1 (FN1), Metallothionein 1F (MT-1F), and Trefoil Factor 3 (TFF3) (19). Differences in study populations and research objectives are the primary reasons for variations in model composition, which in turn lead to disparities in clinical application value.

In the present study, we focused specifically on clinically low-risk PTMC to highlight the clinical relevance of occult CLNM. We further confirmed that sex, age, and tumor size were independent risk factors for CLNM. Our findings revealed a consistent inverse linear relationship between age and risk of CLNM, which is consistent with the findings of previous studies (24). Similarly, tumor size had a positive linear relationship with the risk of CLNM, confirming that greater tumor size was directly associated with an elevated risk of CLNM. HT is considered a protective factor against tumor progression (25), and our previous study demonstrated that it is negatively associated with lymph node metastasis in patients with PTMC (26). However, in this study, it was not selected as a significant independent predictive factor in the final model. Tumor location is also a significant factor that influences lymph node metastasis in thyroid cancer. For example, tumors in the upper portion are associated with a greater risk of lateral neck and skip metastases, whereas tumors in the lower portion of the thyroid are more likely to develop CLNM (27). In our study, compared with tumors in the upper portion, tumors in the middle (OR 1.68, 95% CI 1.36–2.09; p < 0.001) and lower (OR 2.82, 95% CI 2.22–3.57; p < 0.001) portions were associated with a significantly increased risk of CLNM. Overall, the risk factors identified in our model are consistent with those reported in the literature. Notably, these factors are clinical variables that can be easily evaluated during routine outpatient consultations.

In the current study, the predictive model exhibited a diagnostic performance with an AUC of 0.700. Despite this moderate discriminative ability, decision curve analysis supported its potential clinical utility. When determining the appropriate treatment strategy, it is essential to inform patients about the risk of occult CLNM and clearly delineate the advantages and disadvantages associated with each available therapeutic option, including surgery, AS, and RFA. Thus, this parsimonious model may provide auxiliary clinical value for outpatient consultation. It can aid clinicians in patient risk communication and facilitate preliminary risk stratification and clinician–patient communication for patients with clinically low-risk PTMC.

It is noteworthy that the AUC of 0.700 in our model is slightly lower than those reported in recent models. Zhang et al. (2026) reported an AUC of 0.768 for their CLNM prediction model incorporating additional ultrasonographic features (17), and Lin et al. (2026) achieved an AUC of 0.803 using a multimodal ultrasound radiomics-based nomogram (18). However, our model utilizes only four easily accessible clinical variables (sex, age, tumor size, and tumor location), which can be evaluated during a standard outpatient consultation without additional imaging analysis or computational complexity.

This study had several important limitations. Its retrospective nature meant that critical methodological elements such as sample size determination and the standardized protocol for preoperative imaging evaluations, especially US, were not prospectively controlled. Consequently, assessments of cervical lymph node status could have been influenced by variability between operators. Although tumors exhibiting evidence of capsular invasion or suspected ETE on US were excluded from the analysis, microscopic ETE was identified in 1,685 patients (40.1%). These findings suggest a potential for considerable selection bias. Additionally, while the presence of CLNM was examined, variables with potentially greater prognostic significance, such as the number of mLNs and presence of ENE, were not analyzed in depth in this study. These limitations underscore the necessity for future research that employs more rigorously designed prospective studies with larger cohort sizes. Recurrence risk was retrospectively reclassified using the 2025 ATA stratification to reduce heterogeneity from iterative guideline updates. However, patients were managed under older guideline versions, and the mismatch between historical practice and modern recurrence risk stratification frameworks represents another limitation. Specifically, among the patients initially screened, some were excluded due to missing data on the features of metastatic lymph nodes, which inevitably affects the retrospective recurrence risk reclassification. However, as the nomogram relies exclusively on preoperative clinical variables, this bias is expected to have minimal impact on model performance.

The nomogram is intended for initial risk communication and patient education during outpatient consultations, and should not be used as the sole basis for choosing treatment. For patients with intermediate- or high-risk, the nomogram should be combined with detailed ultrasound features (such as proximity to the capsule) or, when available, molecular testing before making therapeutic decisions.

In summary, we constructed and validated a clinical nomogram that incorporated four easily accessible clinical variables: sex, age, tumor size, and tumor location. The model demonstrated moderate discriminative ability, robust clinical applicability, and considerable potential to facilitate preliminary risk stratification and clinician–patient communication for patients diagnosed with clinically low-risk unifocal PTMC.

## Data Availability

The raw data supporting the conclusions of this article will be made available by the authors, without undue reservation.
